# Kirigami/origami: unfolding the new regime of advanced 3D microfabrication/nanofabrication with “folding”

**DOI:** 10.1038/s41377-020-0309-9

**Published:** 2020-04-30

**Authors:** Shanshan Chen, Jianfeng Chen, Xiangdong Zhang, Zhi-Yuan Li, Jiafang Li

**Affiliations:** 1 Key Lab of Advanced Optoelectronic Quantum Architecture and Measurement (Ministry of Education), Beijing Key Lab of Nanophotonics & Ultrafine Optoelectronic Systems, and School of Physics, Beijing Institute of Technology, 100081 Beijing, China; 2 College of Physics and Optoelectronics, South China University of Technology, 510640 Guangzhou, China

**Keywords:** Micro-optics, Nanophotonics and plasmonics

## Abstract

Advanced kirigami/origami provides an automated technique for modulating the mechanical, electrical, magnetic and optical properties of existing materials, with remarkable flexibility, diversity, functionality, generality, and reconfigurability. In this paper, we review the latest progress in kirigami/origami on the microscale/nanoscale as a new platform for advanced 3D microfabrication/nanofabrication. Various stimuli of kirigami/origami, including capillary forces, residual stress, mechanical stress, responsive forces, and focussed-ion-beam irradiation-induced stress, are introduced in the microscale/nanoscale region. These stimuli enable direct 2D-to-3D transformations through folding, bending, and twisting of microstructures/nanostructures, with which the occupied spatial volume can vary by several orders of magnitude compared to the 2D precursors. As an instant and direct method, ion-beam irradiation-based tree-type and close-loop nano-kirigami is highlighted in particular. The progress in microscale/nanoscale kirigami/origami for reshaping the emerging 2D materials, as well as the potential for biological, optical and reconfigurable applications, is briefly discussed. With the unprecedented physical characteristics and applicable functionalities generated by kirigami/origami, a wide range of applications in the fields of optics, physics, biology, chemistry and engineering can be envisioned.

## Materials: An unfolding transformation of the material world

Chinese scientists have conducted a comprehensive review of a method that uses techniques borrowed from origami and kirigami to fabricate tiny three-dimensional (3D) devices. The fabrication of 3D microscale/nanoscale materials holds the key to developing devices with unique physical, optical and electrical properties, including mechanical flexibility and reconfigurability, that do not manifest in their two-dimensional (2D) counterparts. Now, Jiafang Li and colleagues from Beijing Institute of Technology in China, working with Zhi-Yuan Li’s group from South China University of Technology, have reviewed the latest developments in a method that uses techniques from kirigami and origami, such as folding, bending, and twisting to enable the transformation of 2D-to-3D microstructures/nanostructures. The method could lead to the development of novel devices for use in a range of applications, from physics and biology to chemistry and engineering.

## Introduction

Three-dimensional (3D) microfabrication/nanofabrication holds the key to building a large variety of microscale/nanoscale materials, structures, devices, and systems with new, better, and flexible optical, thermal, acoustic, electric, magnetic, and mechanical functionalities compared with their macroscopic counterparts and two-dimensional (2D) planar counterparts^1–3^. Even in the explosively growing areas of 2D materials, for example, the recent demonstration of graphene kirigami^4^ and origami^5^ has opened a new dimension of material engineering promising for unconventional electronic, mechanical, and optical properties such as superconductivity triggered by “magic” twisting^6^. In fact, 3D microfabrication/nanofabrication is so important that it has exerted a dramatic impact on the direction of many research fields. In photonic areas, for instance, the momentum for research on 3D photonic crystals and 3D metamaterials at optical frequencies has largely been weakened in the past decades, mainly due to the challenges in traditional 3D nanofabrication^7^. Although the emerging 2D planar metasurfaces have avoided fabrication difficulties^8–11^, recent advances in device-level integration and reconfiguration (such as metasurfaces integrated with micro-electromechanical systems and spatial light modulators)^12–14^ have once again led to an urgent need for functionality expansion in the third dimension.

While numerous cutting-edge studies have emphasized the necessity and significance of 3D configurations, traditional on-chip 3D microfabrication/nanofabrication techniques rely mostly on a few top–down (subtractive manufacturing) and bottom–up (additive manufacturing) strategies, such as layer-by-layer lithography/stacking^15^, 3D translational writing^16^, and their combinations. Although very mature, highly precise, and widely compatible, these techniques are now approaching the bottleneck of fundamental law limits. In sophisticated complementary metal–oxide–semiconductor (CMOS) techniques, for example, the miniaturization of functional transistors is approaching the physical limit, which is restricted not only by the resolution of deep ultraviolet (UV) lithography but also by the finite size of silicon atoms and lattices. Moreover, all these 3D techniques follow a “linear” rule, i.e. the fabrication volume grows linearly with the fabrication time. This imposes fundamental constraints on both fabricated geometries and the fabrication efficiency, which in turn limit the inspiration for the exploration of new nanomanufacturing platforms.

With the aforementioned considerations, scientists have recently explored some very different 3D fabrication strategies, such as kirigami and origami that make use of the science of cutting and folding flat objects to create versatile 3D shapes^17–19^. Compared with the traditional assembly of isolated objects, such new methodologies enable continuous and direct 2D-to-3D transformations^20^ through folding, bending, and twisting with which the occupied space can vary “nonlinearly” by several orders of magnitude in contrast to the conventional 3D fabrication techniques. More importantly, this new concept of the kirigami/origami technique provides an extra degree of freedom in creating unprecedented 3D geometries beyond the imaginable designs of conventional subtractive and additive fabrication. Therefore, kirigami/origami and related techniques have found emerging applications, such as for deployable devices in the space industry^21^, microelectromechanical/nanoelectromechanical systems^22^, energy storage systems^23^, biomedical devices^24^, and mechanical and photonic materials^25–27^. Especially, the recent progress in nano-kirigami/nano-origami for graphene^4,5^ and chiroptical complexes^28,29^, for example, have opened up promising new avenues for mechanical, electronic, magnetic, and optical applications in the nanoregime.

Aiming to shine a light on this new regime of advanced 3D microfabrication/nanofabrication, this review introduces the latest kirigami/origami-like 3D fabrication at the microscale/nanoscale. Various stimuli of kirigami/origami, such as capillary forces, residual stress, mechanical stress, and responsive forces, and their working mechanisms are briefly introduced. As an instant and direct method, ion-beam irradiation-based nano-kirigami is highlighted in particular. Their capability for reshaping the emerging 2D materials, as well as the potential in biological, optical, and reconfigurable applications, are further discussed and summarized. The opportunities, challenges, and future applications of kirigami/origami-based 3D microfabrication/nanofabrication are discussed. It should be mentioned that, given the large range of scales achievable by kirigami/origami, we choose to confine the scope of this article to structures with scales <1 mm. Studies on relatively large mesoscopic and macroscopic origami/kirigami-type manufacturing can be found in other relevant reviews^1,19^.

## State-of-the-art kirigami/origami at the microscale/nanoscale

**Fig. 1:**
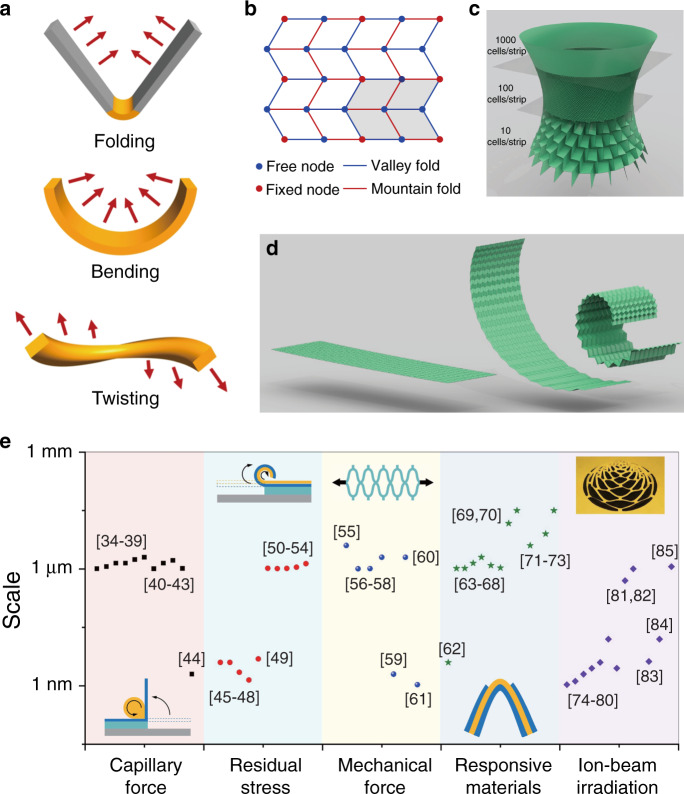
Overview of some typical kirigami/origami strategies. **a** Schematic illustrations of folding, bending and twisting. **b** Mountain/valley fold orientations and patterns of fixed/free nodes in a Miura-ori pattern^31^. **c** Hyperboloid constructed by employing different densities of facets. **d** Generalized cylindrical Miura-ori patterns^31^. **e** Statistics of the scale and the type of stimulus employed in reported 3D microscale/nanoscale kirigami/origami techniques^34–85^. **b**–**d** Reprinted with permission from ref. ^31^

General origami (also named paper-folding) starts from a continuous flat object, and a 2D-to-3D transformation is enabled by the “folding” process. In contrast, kirigami (also named paper-cutting) includes both the processes of “cutting” and “folding”. However, in many studies, the researchers did not count the pre-patterning/lithography procedures, which resulted in no clear boundary between origami and kirigami in many works. Nevertheless, “folding” is the most common characteristic of both origami and kirigami, for which the basic actions can be simply sorted into three types, as illustrated in Fig. 1a. The first is rigid folding of subunits along a flexible hinge^20^, where deformation occurs. The second scheme is gradual bending^20^, in which the whole subunit is deformed. The third type is the multidirectional twisting^30^ that involves folding or bending actions in opposite directions, which is not applicable to traditional 3D fabrication.

At first glance, the basic deformations seem very simple, and the shape transformations have been treated as self-assembly due to the easily predictable targets. However, for more advanced designs in which multiple deformations are highly interlinked, the final structural formation has many variables and is sometimes unpredictable without an understanding of the fundamentals. Moreover, different from translational microfabrication/nanofabrication, these operations can result in modification of the occupied space by several orders of magnitude. For example, as depicted in Fig. 1b–d, by carefully designing the mountain/valley orientations, the pattern of fixed/free nodes and their densities, complicated hyperboloid and cylindrical Miura-ori patterns can be created^31^. It should be mentioned that such kirigami/origami methodologies have been rapidly developed in recent years, leading to advanced design rules in graphene kirigami^32^ and lattice kirigami^33^. In comparison, experimental demonstrations at the microscale/nanoscale lag far behind the corresponding mathematical and physical models. Therefore, the practical development of folding, bending, and twisting strategies at desired locations and in desired directions is highly desirable to achieve advanced microscale/nanoscale kirigami/origami, which will be introduced in the following.

## Microscale/nanoscale kirigami/origami with prescribed patterns

On the microscale/nanoscale, the kirigami/origami processes cannot be directly accomplished by hand or with macroscopic tools due to the limited space. Instead, they usually occur in an indirect manner, i.e. planar patterns are first systematically prescribed, and transformations are subsequently triggered by some “indirect” forces. Among various strategies, in Fig. 1e, we summarize several “indirect” mechanisms or techniques used as the stimuli of kirigami/origami, such as capillary forces^34–44^, residual stress^45–54^, mechanical forces^55–61^, responsive materials^62–73^, and ion-beam irradiation stress^74–85^. These stimuli provide the basic forces or stress *σ*(*r*) to drive the objects into a new equilibrium state, where both the net force $F = {\int}_0^R {\sigma \left( r \right){\rm{d}}r}$ and the net moment $M = {\int}_0^R {r\sigma \left( r \right){\rm{d}}r}$ are equal to zero^85^. Therefore, the abrupt distribution of the driving stress determines the fabrication resolution, with values from a few tens of nanometres to several hundreds of micrometres widely achievable, as shown in Fig. 1e.

## Origami induced by capillary forces and residual stress

**Fig. 2:**
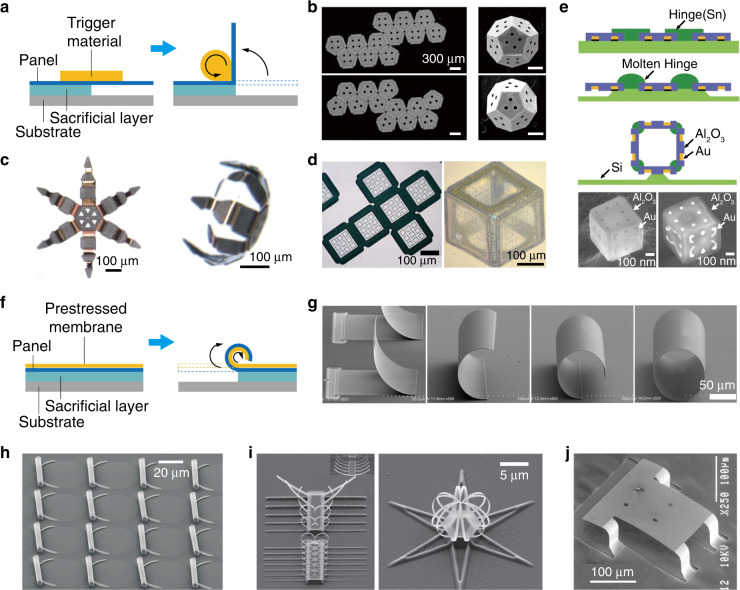
Origami induced by capillary forces and residual stress. **a** Schematic illustration of the capillary force and examples: **b** metallic dodecahedra^40^; **c** reversible actuation microgripper^39^; **d** cubic structure with “C-shaped” split-ring resonators (SRRs) patterned on each window^43^; and **e** self-folded polyhedral geometries with multilayer patterned structures, along with SEM images^41^. **f** Schematic illustrations of residual stress. **g** Rolled-up bilayer structures with different turns^52^. **h** Array of rolled-up nanomembranes^49^. **i** Ion-beam-induced plastic deformation^74^. **j** Standing microstage with bending hinges^88^. Images reprinted with permission from: **b** ref. ^40^ from NAS Publishing; **c** ref. ^39^, **d** ref. ^43^, **e** ref. ^41^, **h** ref. ^49^, **i** ref. ^74^ from Wiley; **g** ref. ^52^ from ACS; **j** ref. ^88^ from Elsevier

The surface tension force is one of the most natural stimuli that can trigger 3D shape transformations, such as the folding and curling of flowers and plant leaves^86,87^. In laboratories, capillary forces and residual stress, as two typical surface tension forces, have been widely employed in origami-like shape transformations. As illustrated in Fig. 2a, when the trigger material changes its phase (for example, from a solid to a liquid phase) and reconfigures its shape to minimize the surface energy, the capillary force released at the interface will drive the folding of adjacent panels to produce out-of-plane rotation of the panel, resulting in new 3D structures^86,87^. For example, Pandeya et al. heated a solder to melting, upon which the molten solder shrunk and induced a capillary force^40^. As a result, the suspended panel was pulled up and folded strictly according to the predesigned procedures, as shown in Fig. 2b. To achieve reversible reconfiguration of 3D microstructures/nanostructures, Randhawa et al. proposed actuating microstructures based on the reversible surface stress during oxidation or reduction of a copper surface^39^. In detail, when chromium/copper bilayers are exposed to oxidative or reductive environments, the oxidation or reduction of the copper surface can alter the curvature of the bilayer, thus producing spontaneous and reversible closing and opening of a micro-paw, as demonstrated in Fig. 2c. One important feature of this type of assembly is that versatile surface patterns can be predesigned on the panels before the folding process, thus forming new classes of functional 3D structures under capillary forces, such as the closed polyhedral structures shown in Fig. 2d–e^41,43^.

In addition, without any change in material phase, residual stresses are commonly induced by the strain mismatch between multilayer materials. As illustrated in Fig. 2f, when the sacrificial layer is removed (e.g. by etching), the suspended panels can self-roll to produce tubular, scroll-like, or polyhedral microstructures due to the release of the residual stresses at the bottom interface^47,48,51–54^. For example, Huang et al. used the dynamic release process of SiNx bilayer membranes based on the strain mismatch to construct self-rolled-up tubular structures^52^, as shown in Fig. 2g. Through strain engineering of SiO/SiO_2_ nanomembranes on polymers, Mei et al.^49^ fabricated integrative and functionalized rolled-up tubes, as shown in Fig. 2h, whose diameters and lengths can be precisely tuned. More flexibly, Chalapat et al.^74^ achieved versatile complex 3D structures with folding radii as small as 10 nm by utilizing reactive ion etching of Ti/Al/Cr film. The physical mechanism originated from the accumulation of compressive stress induced by the reactive ion etching, which relaxed and stimulated bending of the structure, as shown in Fig. 2i. In other designs, Vaccaro et al. developed a valley- and mountain-fold method to employ two types of hinges (called tani-ori and yama-ori) for the same epitaxial layers to build standing microstages^88^, as shown in Fig. 2j.

## Kirigami triggered by mechanical stress and substrate engineering

**Fig. 3:**
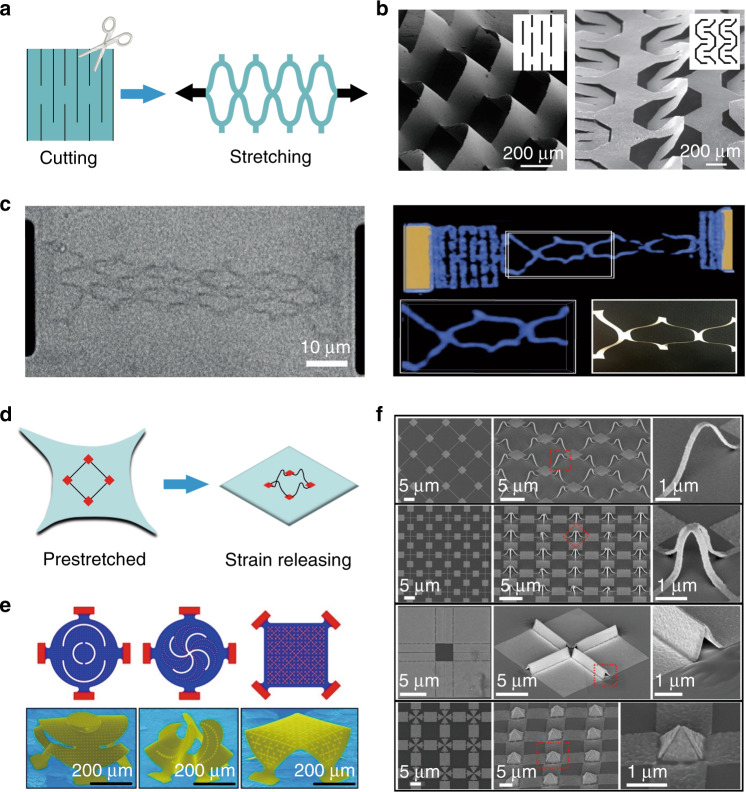
Kirigami triggered by mechanical stress and substrate engineering. **a** Schematic illustration of the stretching-induced force. **b** Kirigami patterns in elastic nanocomposites after photolithography^55^. Insets show the corresponding 2D designs. **c** Graphene kirigami^4^. **d** Schematic illustration of the strain release-induced force. **e** Mechanically buckled 3D mesostructures^56^. **f** Mechanically assembled structures formed by the release of a biaxially deformed polydimethylsiloxane substrate^61^. Images reprinted with permission from: **b** ref. ^55^, **c** ref. ^4^; **e** ref. ^56^ from NAS Publishing; **f** ref. ^61^ from ACS

Mechanical stress and substrate engineering represent another type of approach for kirigami. As illustrated in Fig. 3a, the scheme with mechanical stress employing indirect cutting and stretching of customizable microstructures/nanostructures is very similar to macroscopic paper cutting. In this case, Shyu et al. created kirigami patterns in nanocomposites by photolithography^55^, which could be stretched after the nanocomposite sheet was detached from the substrate. Consequently, the elasticity was engineered by defect patterning, as shown in Fig. 3b. Similarly, Xu et al. used this principle to develop a kirigami nanocomposite for use as wide-angle diffraction gratings^89^. More precisely, Blees et al. successfully applied this method to graphene kirigami and constructed stretchable graphene in solution with outstanding mechanical and optical properties^4^. As shown in Fig. 3c, the graphene spring was stretched by approximately 70% (left diagram), and the 3D reconstruction figures show good consistency between the graphene kirigami and paper cutting results (insets of the right diagram).

Substrate engineering can also be employed for 3D shape transformation. As schematically illustrated in Fig. 3d, when 2D precursors are partially fixed on a planar prestretched substrate, compressive forces can be driven by the movable bonding sites during the release of the substrate, which then induce well-defined buckling to form versatile 3D structures^56^. In this aspect, Zhang et al.^56^ developed very sophisticated techniques, as well as advanced theoretical designs, with which various buckling and twisting of morphable 3D mesostructures have been achieved (Fig. 3e)^90^. More recently, Liu et al. developed a metal-assisted transfer strategy on an elastic substrate and fabricated high-resolution nanostructures with metal gaps of sub-10 nm^61^, as shown in Fig. 3f.

## Origami using responsive forces

**Fig. 4:**
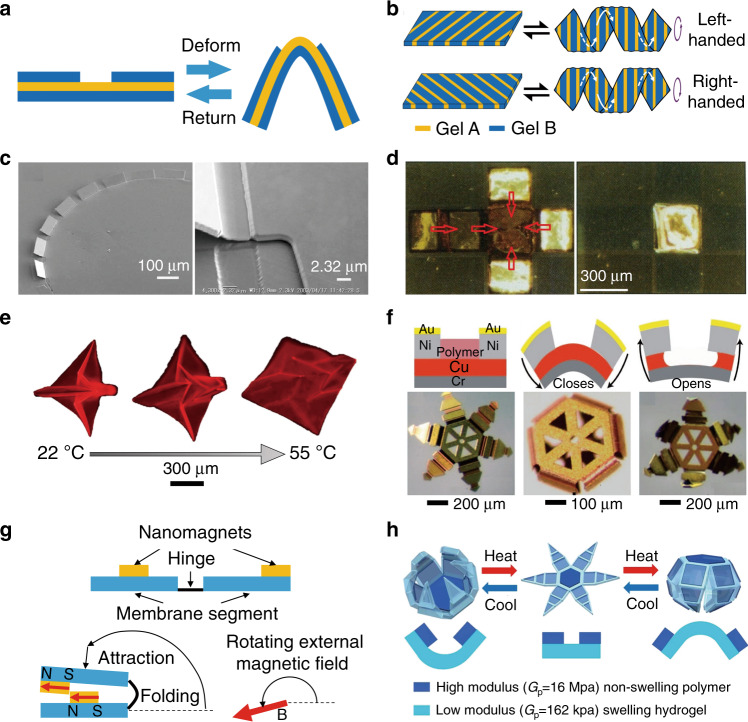
Origami induced by responsive forces. **a** Schematic illustrations of the forces arising from active material-induced physical or chemical reactions. **b** Schematic illustration of the self-shaping helical structures^91^. **c** Micro-origami by optical activation^64^. **d** Electrically controlled and reversible self-folding box^67^. **e** Reversibly self-folding origami with micropatterned photo-crosslinkable polymers^72^. **f** 3D microgrippers based on the chemical reaction of polymer and Cu layers with acetic acid and hydrogen peroxide^65^. **g** Magnetically activated folding of membranes^62^. **h** Thermo-magnetically responsive microgrippers^73^. Images reprinted with permission from: **c** ref. ^64^, **g** ref. ^62^ from Elsevier; **d** ref. ^67^ from AAAS; **e** ref. ^72^ from Wiley; **f** ref. ^65^, **h** ref. ^73^ from ACS

Physical or chemical reactions in active materials can induce differential responsive forces between the interfaces of multilayered structures, which can trigger 3D shape transformations similar to the case of residual stress, as illustrated in Fig. 4a. For example, Wu et al. designed a fibre-like hydrogel sheet with two gels of different shrinkage and elastic moduli and achieved planar-to-helical 3D shape transformation by varying the concentration of NaCl solution due to the chemical difference of the hydrogels^91^ (Fig. 4b). Optical actuation was achieved by Ocampo et al. by shining a laser on mirrors fabricated on an InGaAs layer, which was possibly triggered by the stress due to photogenerated carriers^64^ (Fig. 4c). Electrically reversible 3D transformation of rigid plates was demonstrated by Smela et al. through the design of a hinge consisting of polymer and gold bilayers^67^ (Fig. 4d). Moreover, thermally photo-crosslinkable copolymers^72^ (Fig. 4e) and chemical reaction of polymer/Cu layers can also be employed for folding and unfolding of 3D microstructures^65^ (Fig. 4f).

In addition to the engineering of locally active materials to induce desirable transformations, magnetic forces can trigger a remote transformation by applying an external magnetic field. As illustrated in Fig. 4g, when an external field magnetizes nanomagnets, rotation of the external magnetic field can induce a torque and then fold the membrane^62^. For example, Nichol et al. developed two-step magnetic self-folding of structural arrays with accurate alignment^62^. Moreover, Breger et al. embedded iron oxide (Fe_2_O_3_) nanoparticles into porous hydrogels with reversible swelling responses^73^, with which 3D origami was flexibly triggered by varying the thermal and pH environments or external magnetic field (Fig. 4h).

## Instant nano-kirigami based on focussed-ion-beam (FIB) irradiation

**Fig. 5:**
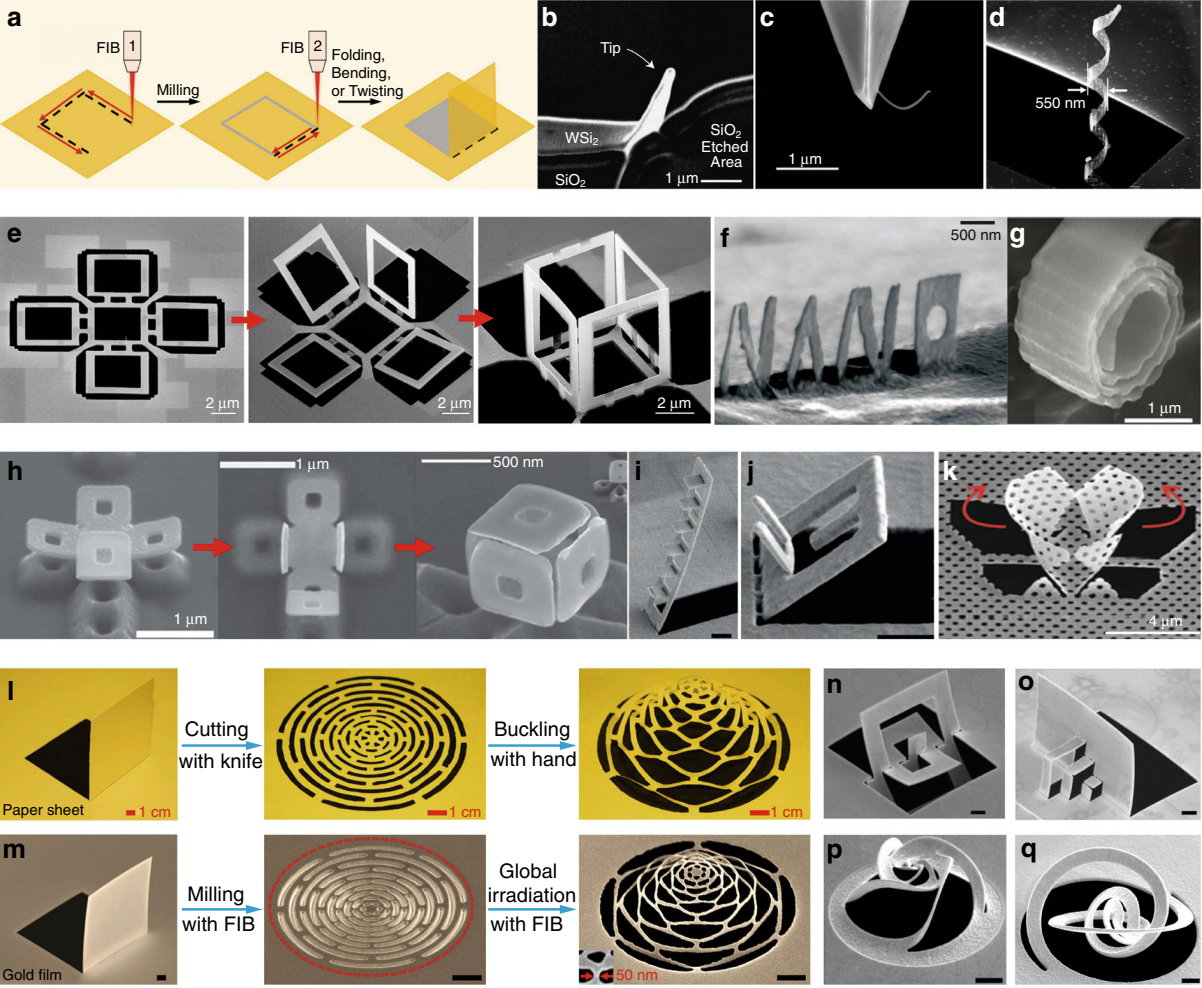
Kirigami based on ion-beam irradiation. **a** Schematic illustration of the fabrication scheme with an FIB. **b**–**k** SEM images of various 3D structures fabricated by tree-type folding/bending with an FIB^30^. **b** Folded emitter tip^76^. **c** Curved carbon nanotube^92^. **d** Nanohelix^79^. **e**, **h** Microcubic structures^74,79^. **f** Nanoscale script^82^. **g** Swiss roll^81^. **i**, **j** Staircase-like and composite U-shaped structures^80^. **k** Twisting butterfly wings^94^. **l** Camera images of paper kirigami and **m** SEM images of close-loop nano-kirigami of an expandable dome^28^. **n**–**q** 3D structures fabricated by close-loop nano-kirigami^28,30^. Scale bars of **i**, **j**, **m**–**q**: 1 µm. Images reprinted with permission from: **b** ref. ^76^; **c** ref. ^92^, **g** ref. ^81^, **h** ref. ^74^ from Wiley; **d**, **e** ref. ^79^; **f** ref. ^82^ from IOP; **i**, **j** ref. ^80^ from Springer Nature; **k** ref. ^94^ from RSC

One challenge of traditional kirigami/origami with prescribed patterns is that most of the schemes involve multiple materials or multistep processes that have to be accurately planned, making it difficult to instantly add/remove desirable components on-site. Interestingly, recent studies have shown that FIBs not only have the functions of imaging, etching, and auxiliary deposition but also can induce desired stresses in suspended nanostructures. By instantly cutting and folding suspended nanomembranes with high resolution, the basic actions of kirigami can be achieved, such as folding, bending, and twisting, making an FIB an ideal tool for instant nano-kirigami^28^. As shown schematically in Fig. 5a, this maskless processing of microstructures/nanostructures is accomplished by programming ion-beam irradiation in two sequences, i.e. high-dose FIB milling and subsequent low-dose irradiation of the designed areas. The basic principle is that, when a thin film is exposed to high-energy ion irradiation, strong physical bombardment will introduce vacancies, ion dopants, atom dislocation, redeposition, etc., which exert significant effects on the local stress, strain, or deformation of the thin film^30^.

Undesirable stress, surface damage, and implantation induced by an FIB occur in the nanofabrication process and have long been avoided as much as possible. Nevertheless, in 2005, Yoshida et al. made use of this so-called drawback and demonstrated the early phenomenon of structural bending with an FIB (Fig. 5b)^76^. Park et al. produced curved carbon nanotubes in 2006 using the same method^92^. At the same time, Xia et al.^79^ demonstrated the high flexibility, controllability, and repeatability of 3D nanofabrication with FIB-induced stress by fabricating complex 3D nanohelices (Fig. 5d) and assembled cubic frames (Fig. 5e). This important demonstration stimulated intensive studies by other researchers^85,93^, and versatile 3D nanostructures were manufactured (Fig. 5f–k)^80,82,94^, among which the first photonic application was demonstrated by Cui et al. in 2015 through folding of a vertical split-ring resonator (SRR) array on a metallic hole array^80^. However, most of these nanostructures were fabricated by local irradiation of specific areas, which induced zero curvature except at the folding regions, failed in simultaneous deformation of multiple subunits, and suffered from overhead beam-blocking effects^28^. Furthermore, from the viewpoint of topological classification, these transformations belong to the tree-type multibody system^30^, in which the relative motion of each subunit is independent. As a result, the flexibility, geometry, and functionality of the structural transformation are largely limited.

Inspired by a traditional Chinese paper-cutting art named “pulling flower” (depicted in Fig. 5l), in 2018, we demonstrated a close-loop nano-kirigami method^28^. As illustrated in Fig. 5m, by employing an FIB instead of knives/scissors to cut a precise pattern in a free-standing gold nanofilm and using the same FIB irradiation instead of hands to gradually “pull” the 2D pattern, a complex 3D shape was formed in a single fabrication system. The key difference from other methods is that the relative transformations of each subunit within the interlinked loop are dependent, i.e. a relative deformation of one component affects the relative changes of the others. Therefore, by deliberately designing lithographed 2D patterns, the stress distribution within the nanofilms can be readily engineered during FIB irradiation. Under the topography-guided stress equilibrium, versatile 3D shape transformations, such as upward buckling, downward bending, complex rotation, and twisting of nanostructures, can be precisely achieved with resolutions down to sub-50 nm, representing the intrinsic features of nano-kirigami. As a result, unprecedented 3D nanogeometries have been directly and instantly achieved by FIB-based close-loop nano-kirigami, as shown in Fig. 5n–q.

From the above discussions, it can be seen that capillary forces, residual stress, and responsive forces can induce free-form 2D-to-3D transformations. However, these techniques are normally based on multiple materials and operate at a relatively slow speed. While kirigami using mechanical stress and substrate engineering is very straightforward and has good stretchability, it relies on relatively large actuators or elastic substrates. Meanwhile, the origami with magnetic forces is very appealing in swift transformations, but the spatial resolution and multidirectionality of the deformations need further improvement. In comparison, FIB-based nano-kirigami provides instant, direct, and on-site 2D-to-3D transformations with the highest spatial resolution, holding promise for nanoscale device applications. However, the currently reported methods are based on metallic nanofilms, and the fabrication speed with an FIB is relatively slow for large-scale applications. Therefore, the existing kirigami/origami at the microscale/nanoscale still needs substantial improvement for device-level applications. Nevertheless, our preliminary studies have shown that FIB-based nano-kirigami methods can be applied to a wide variety of metallic and dielectric materials, including aluminium, silver, silicon, silicon nitride, etc. (results not shown). Furthermore, the nano-kirigami strategy is also applicable to general metal–insulator–semiconductor and silicon-on-insulator platforms. In addition, to meet the demand for fast and large-scale fabrication, one solution is to combine the nano-kirigami principle with electron-beam lithography (EBL), standard UV lithography, or CMOS techniques, which is under investigation.

## Potential applications

Advanced kirigami/origami provides a facile and easily accessible approach for modulating the mechanical, electrical, magnetic, and optical properties of existing materials. Different from the mechanical applications of the mesoscopic counterparts, the microscale/nanoscale folding, bending, twisting, stretching, and reconfiguration in kirigami/origami have extensive potential for the reshaping of 2D materials, as well as in biological, optical, and reconfigurable applications.

## Reshaping 2D materials

Emerging 2D materials, such as graphene and MoS_2_, have been extensively studied owing to their extremely thin thickness and extraordinary electronic, optical, and mechanical properties. Very recently, researchers have extended the concept and techniques of kirigami/origami to the regime of 2D materials^4,5,95–99^, which has created an effective and promising platform to reshape 2D monolayers into 3D architectures through precise patterning, bending, folding, and twisting. Such 2D-to-3D transformation can enable unique properties very different from those of the original 2D materials, such as extremely large stretchability^99^, reversible photoresponsiveness^97^, and greatly enhanced light–matter interaction^96^. Moreover, it creates new opportunities for further development of self-actuated functional devices that may respond to mechanical forces, light or magnetic fields, thermal variations, or chemical modifications.

**Fig. 6:**
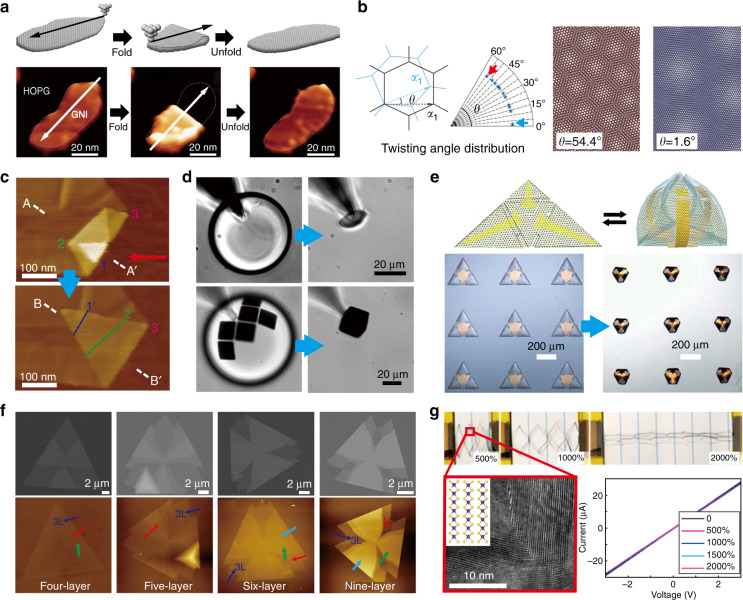
Reshaping 2D materials. **a**–**c** Graphene origami: **a** folding and unfolding a graphene piece along the predefined direction^5^; **b** bilayer graphene-based nanostructures with controllable twist angles^5^; **c** Z-shaped self-folded graphene segment^95^. **d**, **e** MoS_2_ origami: **d** cube-shaped self-folded structure^96^; **e** reversible self-folding pyramids^97^. **f** WSe_2_ kirigami^98^: SEM micrographs and AFM images of differently folded WSe_2_ layers. **g** PtSe_2_ kirigami^99^. Images reprinted with permission from: **a**, **b** ref. ^5^ from AAAS; **c** ref. ^95^; **d** ref. ^96^, **e** ref. ^97^, **f** ref. ^98^, **g** ref. ^99^ from ACS

For example, in 2015, Blees et al. applied kirigami principles to graphene sheets to build interesting mechanical metamaterials, such as stretchable graphene transistors, out-of-plane pyramidal springs, and remotely actuated graphene devices^4^. These results successfully established graphene kirigami as a customizable approach for fashioning atomically thin graphene sheets into complex 3D multifunctional devices controllable by magnetic and optical fields. More recently, atomically precise graphene origami was demonstrated by Chen et al. based on an advanced scanning tunnelling microscopy (STM) technique^5^, as shown in Fig. 6a, b. In this method, the STM tip was used to lift a graphene layer by the edge, drag the graphene along the predetermined direction, and release the moving portion of the graphene at the desired location. Such precise manipulation enabled twisting of bilayer graphene with nearly arbitrary angles (Fig. 6b), which may generate emerging bilayer graphene with a magic twist angle^6^. Similarly, an atomic force microscopy (AFM) tip can also be used for origami purposes. As demonstrated in Fig. 6c, a Z-shaped self-folded graphene segment can be transformed back into a flat membrane by using the AFM technique^95^, offering an effective strategy in the pursuit of reversible graphene devices.

In addition to graphene, other promising 2D materials, such as MoS_2_, WSe_2_, and PtSe_2_, have also been reshaped into 3D structures by using kirigami/origami principles. As shown in Fig. 6d, Reynolds et al.^96^ demonstrated that a MoS_2_ monolayer can be folded into 3D shapes by the capillary force induced by the surface tension of a droplet. They further designed rigid metal panels connected by MoS_2_ hinges to achieve a self-folded cube-shaped microstructure (bottom of Fig. 6d). At the same time, Xu et al.^97^ fabricated reconfigurable and optically active 3D MoS_2_ micropyramids by patterning monolayer MoS_2_ and gold onto differentially photo-crosslinked polymeric thin films, as shown in Fig. 6e. With this method, the optical detection areas in these microstructures are highly tuneable. Moreover, Cai et al.^98^ reported novel kirigami structures of multilayered WSe_2_ formed by a simple chemical vapour deposition and etching method. The scanning electron microscopic and AFM images in Fig. 6f clearly show the advanced kirigami of WSe_2_ heterostructures from four to nine layers. In device applications, Okogbue et al.^99^ recently developed a novel electrical conductor based on metallic 2D PtSe_2_/PI kirigami structures, which exhibited an extremely large stretchability of ∼2000% without compromising their intrinsic electrical conductance (Fig. 6g). These structures further showed a strain-tuneable and reversible photoresponse when interfaced with semiconducting carbon nanotubes.

The representative examples discussed above, as a portion of the work in this area, clearly demonstrate the feasible incorporation of kirigami/origami with versatile 2D materials, as well as the resulting unconventional and tailorable 3D geometries and configurations. These 3D atomic, nanoscale, or microscale structures can show a wide variety of novel optical, electrical, mechanical, chemical, and biological properties or functionalities that are not easy, or even impossible, to achieve using traditional microfabrication/nanofabrication technologies. Therefore, kirigami/origami provides a novel platform for studying and exploring the rich multiphysical properties of 2D materials, which may offer vast opportunities for emerging electronics and optoelectronics.

## Biological applications

**Fig. 7:**
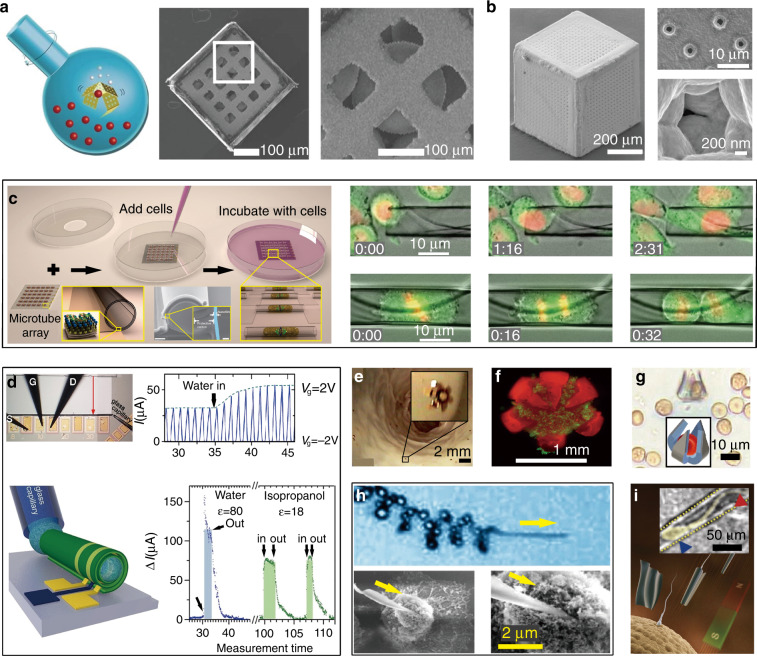
Potential biological applications. **a** Self-loading of microcontainers filled with an aqueous suspension of glass beads^36^. **b** Self-folded microcontainers with structured nanopores on the walls^42^. **c** Self-rolled-up functionalized microtube device for single-cell studies^53^. **d** Chemical sensing of solvents with different polarities^100^. **e** μ-Grippers covering the colon surface to sample biological tissue^101^. **f** Optical and fluorescent images of a detached theragripper tightly gripping a clump of cells^102^. **g** Red blood cells captured by the self-folding of single-cell grippers^103^. **h** Self-propelled nanotools moved by bubble propulsion^104^. **i** Sperm-flagella-driven micro-bio-robot^105^. Images reprinted with permission from: **a** ref. ^36^ from RSC; **b** ref. ^42^, **e** ref. ^101^ from Elsevier; **c** ref. ^53^, **d** ref. ^100^, **g** ref. ^103^, **h** ref. ^104^ from ACS; **f** ref. ^102^, **i** ref. ^105^ from Wiley

A number of folded, curved, and twisted structures have been assembled by novel kirigami/origami techniques for versatile biomedical devices. As early as 2008, Leong et al.^36^ demonstrated mass-producible, mobile, three-dimensionally patterned microcontainers fabricated by utilizing thin-film residual stresses. These microcontainers load themselves as they self-assembled from cruciform templates at approximately 40 °C and can be used to encapsulate both non-living and living objects. Different from traditional studies on cell biology performed with 2D cell cultures, such as in Petri dishes and on patterned planar substrates, these microcontainers have porous walls and interact with their surroundings in all three dimensions, as shown in Fig. 7a. Furthermore, the porous microcontainers can be utilized for advanced cell encapsulation where the pores in all three dimensions can be engineered to control the diffusion of certain materials to the encapsulated cells. For example, Randall et al.^42^ demonstrated that the nanopores on the walls can be utilized to inhibit the diffusion of immune components and permit the adequate delivery of insulin, providing the possibility of developing a lithographically structured bioartificial pancreas by employing the microcontainers (Fig. 7b). Because many cellular processes occur in spatially confined physiological environments and the cellular behaviour and function can be greatly dictated by limiting the interactions with the surroundings, Xi et al.^53^ developed a microtubular platform that served as a 3D tissue culture scaffold to investigate the division of living mammalian cells under tubular confinement, as shown in Fig. 7c. The 3D single-cell cavity platform formed by origami based on responsive forces allowed for non-invasive encapsulation of different types of individual mammalian cells. Meanwhile, by utilizing the kirigami/origami method, large-area devices can be readily compacted to footprints orders of magnitude smaller than the original planar structure. For example, 3D tubular inorganic thin-film transistors with bending radii of <5 μm have been constructed by Grimm et al. (Fig. 7d)^100^, which naturally offer a microfluidic channel with intriguing potential for future in situ chemical and biological sensing applications.

Moreover, benefitting from their small scales and high mobility, origami-like untethered microgrippers have been successfully developed for biologic tissue sampling (Fig. 7e)^101^, delivery and release of drugs (Fig. 7f)^102^, and capture of single red blood cells (Fig. 7g)^103^. In addition, the devices produced from origami-induced self-rolled-up microtubes have been used to perform self-propelling tasks, such as catalytic micromotors that enable tubes to drill and embed themselves into biomaterials (Fig. 7h)^104^ and a micro-bio-robot that can be guided to defined positions (Fig. 7i)^105^.

## Optical applications

**Fig. 8:**
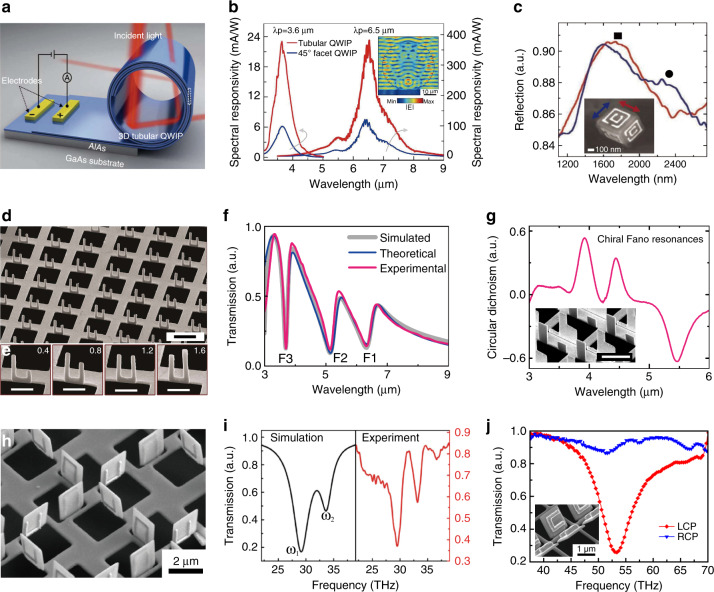
Optical applications. **a**, **b** Broadband and enhanced responsivity of 3D tubular QWIPs^54^. **c** Single-particle Fourier transform infrared (FTIR) reflection measurement of a cubic structure with double optically active SRRs patterned on all faces^41^. **d**–**f** Fano resonances of surface plasmons in the mid-infrared region^106^. **g** Giant bisignate circular dichroism of a fabricated DPMM^84^. **h**, **i** Toroidal excitation in folded 3D metamaterials^107^. **j** Experimental transmission spectra of chiral folded metasurfaces under the illumination of different circularly polarized waves^78^. Scale bars: **d**, **g** 2 µm and **e** 1 µm. Images reprinted with permission from: **a**, **b** ref. ^54^ from AAAS; **c** ref. ^41^, **h**, **i** ref. ^107^ from Wiley; **j** ref. ^78^ from ACS

One important advantage of kirigami/origami at the microscale/nanoscale is that the resulting structural features are comparable with optical wavelengths, facilitating the generation of useful optical resonances. Meanwhile, compared to the 2D precursors, the flexible 3D microstructures/nanostructures obtained by kirigami/origami can exhibit unique optical properties due to their special geometries. For example, by employing residual stress-enabled origami, Wang et al.^54^ demonstrated 3D tubular quantum well infrared (IR) photodetectors with enhanced responsivity and detectivity, broadband enhanced coupling efficiency, and omnidirectional detection under a wide incident angle of ±70° (Fig. 8a, b). Moreover, self-assembled origami nanostructures can enable abundant optical functionalities by embedding special patterns in their subunits. As depicted in Fig. 8c, Cho et al. utilized multilayer EBL processes to pattern planar thin films and employed a capillary force to achieve spontaneous folding. As a result, cubic structures embedded with SRR patterns were achieved, with clear optical resonances and the desired polarization dependence successfully implemented^41^.

As introduced in the previous section, FIB-based nano-kirigami can introduce versatile 3D nanostructures with exceptional geometries. However, it was not until 2015 that the first photonic application of this method was demonstrated by Cui. et al. through folding of vertical SRRs on metallic hole arrays^80^, where extraordinary Fano resonances with ultra-high refractive index sensitivity were obtained. More importantly, such a configuration supports a new mechanism of 3D plasmonic conductive coupling, with which triple Fano resonances are readily generated with vertically folded asymmetric SRRs (Fig. 8d–f)^106^. Interestingly, the triple plasmonic Fano resonance states can be tailored by changing the asymmetric arms of the SRRs (Fig. 8f), and strong mutual coupling between two Fano resonance states can be easily achieved, resulting in double Rabi splitting of the triple Fano resonances. Such an experimental demonstration of strong interactions among multiple discrete states and one continuum in an optical system, first proposed by U. Fano in 1961, demonstrated the new physics and powerful engineering capabilities of the optical interaction brought about by tree-type nano-kirigami 3D nanofabrication. Moreover, by folding up asymmetric vertical plate-shaped resonators along a planar air hole array, Tian et al. built a 3D double-plate-based chiral metamaterial, as depicted in Fig. 8g, which simultaneously supported fivefold plasmonic Fano resonances and significant bisignate circular dichroism (CD)^84^.

In addition, SRRs with different orientations and variable folding angles can enable excellent flexibility and controllability in the design of exotic 3D optical metamaterials. As shown in Fig. 8h, i, a novel 3D metamaterial with a high-quality-factor toroidal resonance was constructed by FIB-based rigid folding of silicon nitride plates, on which gold SRRs with different open directions were patterned by EBL^107^. More recently, by applying an FIB to fold the constituent components along certain angles, Yang et al. reported a chiral metasurface composed of folded antisymmetric SRRs (Fig. 8j)^78^. Owing to the broken mirror symmetry, prominent coupling occurred between the electric and magnetic dipole resonances and, consequently, spin-selective transmission in the IR region was observed owing to the pronounced intrinsic chirality.

**Fig. 9:**
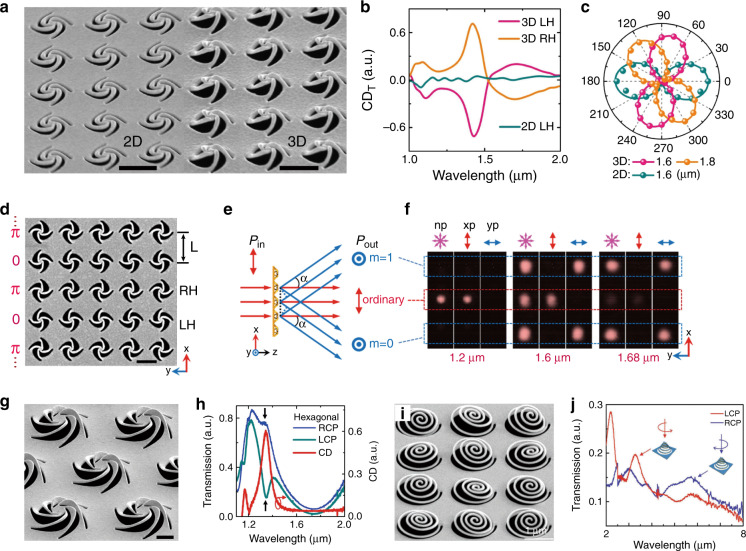
Applications in optical chirality. **a** SEM images of 2D and 3D left-handed (LH) pinwheel arrays^28^. **b** Measured CD spectra for 2D LH, 3D LH, and 3D right-handed (RH) pinwheels^28^. **c** Polar plot of experimental transmission versus detection polarization angle under *x*-polarized incidence for the 2D and 3D LH pinwheels at different wavelengths^28^. **d** SEM image of a linear grating with LH and RH pinwheels fabricated alternately^29^. **e** Schematic of the diffraction properties of the linear grating^29^. **f** CCD camera images of the transmitted light spots at the wavelengths noted under detection with non-polarization (np), *x*-polarization (xp), and *y*-polarization (yp)^29^. **g** SEM image of six-arm pinwheels arranged in a hexagonal lattice^108^. **h** Measured LCP and RCP transmission spectra of three-arm pinwheels arranged in a hexagonal lattice and corresponding CD spectrum^108^. **i** SEM image of a 3D chiral fractal metasurface and **j** its LCP and RCP transmission spectra^109^. Scale bars: 1 μm. **i**, **j** Reprinted from ref. ^109^ with permission from Wiley

Figure 8d–j show that tree-type rigid folding resulted in 3D geometries with relatively large scales. As a result, the operation wavelengths were in the several micrometre or THz region. To further scale down the nanostructures for operation at optical wavelengths, Liu et al. proposed a simple close-loop nano-kirigami method and successfully demonstrated exotic metastructures at telecommunication wavelengths^28^. As shown in Fig. 9a, by carefully designing a 2D spiral pattern and utilizing the nanoscale out-of-plane twisting features of nano-kirigami, 3D pinwheel-like metastructures were readily manufactured. Owing to the parallel electric and magnetic moments induced by the 3D twisted loops, giant intrinsic optical chirality was induced. As plotted in Fig. 9b, c, pronounced CD and giant polarization rotation versus wavelength can be observed in the 3D pinwheel-like metastructures, in strong contrast to the weak phenomenon in the achiral 2D precursors. In particular, the polarization rotation angle reached ~90° at 1.70 µm, corresponding to a recorded large circular birefringence of ~210,000°/mm. More importantly, such close-loop nano-kirigami successfully pushed the operation wavelengths to the telecommunication regime, greatly expanding the application areas of kirigami.

The unique intrinsic chirality of 3D pinwheels can further help in exploring versatile optical functionalities, such as phase and polarization manipulation in metasurfaces. For example, the phase difference between the cross-polarized transmission of left-handed (LH) and right-handed (RH) 3D pinwheels remained constant around *π* over a broad band^29^. Therefore, by alternatively patterning metasurfaces with opposite handedness, an excellent binary diffractive grating for cross-polarized light was readily manufactured, as displayed in Fig. 9d. In this case, when the pinwheel period (*L* = 1.45 µm) is smaller than the operation wavelength *λ*, the transmitted *y*-polarized beams can be diffracted by an angle *α* (Fig. 9e), which was well verified by the recorded diffraction spots (enclosed by blue dashed lines) shown in Fig. 9f. Moreover, owing to the intrinsic phase characteristics of the uniaxial pinwheel structures, a radial diffractive grating was demonstrated by alternatively patterning LH and RH pinwheels in the radial direction, resulting in diffractive spots in the outer rings and the original beam spot in the centre^29^.

With the versatile 3D nanogeometries generated by nano-kirigami, the design and engineering of optical functionalities are greatly enriched. For example, pinwheels with different numbers of arms (Fig. 9g), arranged in square, hexagonal, and honeycomb lattices, were recently systematically investigated^108^. Benefitting from the out-of-plane chiral geometry and lattice symmetry, the physics of Fano resonances can be employed to efficiently enhance the intrinsic 3D CD, which was found to be maximized for three-arm pinwheels arranged in a hexagonal lattice, as shown by the experimental spectra in Fig. 9h^108^. Meanwhile, Tseng et al. reported a 3D chiral fractal metasurface composed of an array of 3D Archimedean spirals produced by FIB-induced deformations (Fig. 9i), which exhibited extraordinary chiral dissymmetry in transmission and broadband near-field chiroptical responses from 2 to 8 µm (Fig. 9j)^109^. With the wide adoption of FIB-based nano-kirigami and related techniques, generation of useful 3D nanogeometries to enrich the field of metasurfaces for the exploration of new physics and novel applications can be envisioned.

## Applications in optical reconfiguration

**Fig. 10:**
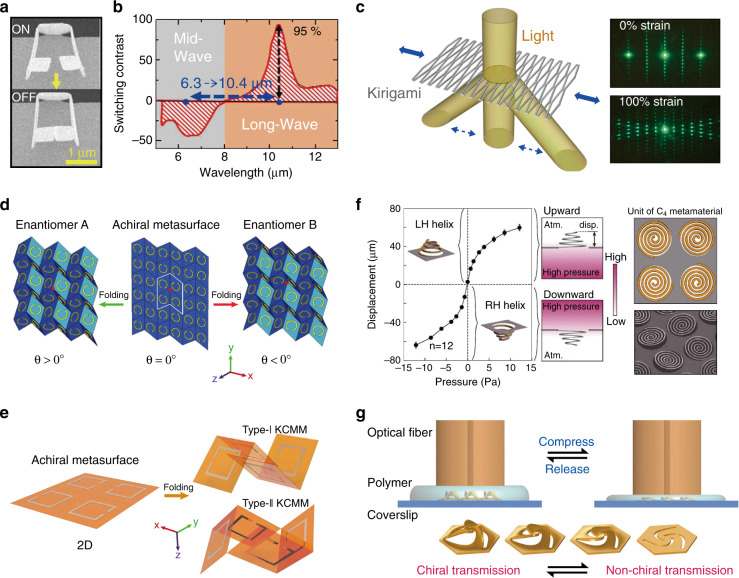
Applications in optical reconfiguration. **a**, **b** Reversible switching of a tuneable 3D SRR metasurface in the infrared (IR) wavelength region^83^. **c** Kirigami nanocomposites as wide-angle diffraction gratings^89^. **d** Schematic of a Miura-ori chiral metamaterial, in which the chirality can be switched by changing the deformation direction^27^. **e** Schematic illustration of (left) the 2D precursor and (right) two 3D kirigami-based metamaterials with opposite chirality^110^. **f** Displacement of the spiral structure with respect to the applied pressure, which deformed the planar spiral to a left-handed or right-handed helix^111^. The lateral size of each spiral is 150 μm. **g** Mechanical compression and release of a three-arm pinwheel array, which changed the height of the pinwheels and enabled reversible switching between chiral transmission and non-chiral transmission^108^. Images reprinted with permission from: **a** ref. ^83^, **b** ref. ^83^, **c** ref. ^89^ from ACS; **d** ref. ^27^ from Wiley; **e** ref. ^110^, **f** ref. ^111^ from Springer Nature

The realization of reliable high-resolution reconfiguration at optical wavelengths is an important topic and remains a great challenge in modern nanophotonics. For example, advances in metasurfaces now face challenges in dynamic optical reconfiguration^12–14^, and the widely commercialized digital micromirror devices (DMDs) suffer from complicated designs associated with large pixels and slow speed. Advanced kirigami/origami techniques can provide an effective strategy for addressing this issue due to the deformable configurations. For example, an electromechanically tuneable meta-atom composed of an array of 3D nanoscale SRRs (Fig. 10a) was fabricated by Mao et al. in 2016 by folding a substrate-free silicon nitride/gold film using an FIB^83^. As shown in Fig. 10a, under an applied electric current of 20 mA, the arms of the folding units gradually embraced each other due to the Joule heat-induced deformation of the double-layer film. As a result, the gap width between the palms of each SRR decreased from 514 nm to 0, switching the device from the “ON” to the “OFF” state, which induced considerable changes in the light–matter interaction. This process was reversible, and the optical behaviour could be dramatically tuned by changing the gap width. As shown in Fig. 10b, electrothermal reversible switching in the IR spectral region was achieved with a high switching contrast of 95%.

Stretchable and deformable kirigami-induced tuneable optics can be helpful for efficient beam steering, as used in light/laser radar (LIDAR/LADAR) components of robotic systems. To this end, Xu et al. utilized kirigami sheets composed of stiff/strong nanocomposites to address the core issue of tuneable diffraction gratings^89^. As shown in Fig. 10c, a slit-based kirigami pattern formed from thin-film nanocomposites (containing high-performance stiff plastics, metals, carbon nanotubes, etc.) enabled reconfigurable optical gratings with a >100% range of period tunability under wide-angle beam steering. In addition, tuneable chirality can be realized by kirigami/origami-based reconfigurable 3D metamaterials^27,110^. For example, Wang et al. reported an origami-based metamaterial with reconfigurable CD via switching of the folding state of Miura-ori SRRs^27^, as shown in Fig. 10d. A high CD of 0.6 was experimentally observed and switched by controlling the deformation direction and kinematics. Importantly, the relative density of the origami metamaterials was dramatically reduced to only 2% of that of the unfolded structure. Similarly, Fig. 10e shows two types of 3D folded metamaterials made from the same 2D precursor, whose toroidal resonances can be switched between non-chiral and chiral states^110^. With a chip-based prototype, Kan et al. demonstrated a handedness-switchable chiral metamaterial for polarization modulation via vertical deformation of a planar spiral by a pneumatic force, as depicted in Fig. 10f^111^. More recently, Liu et al. embedded a three-arm pinwheel metastructure in a polydimethylsiloxane-mediated polymer and mechanically compressed and released the structure with a fibre tip, as shown in Fig. 10g^108^. The vertical height of the twisted pinwheels can be reversibly tailored owing to the good elasticity of the polymer. Consequently, the optical chiral responses of the stereo twists can be dynamically engineered with a high contrast of >50% at telecommunication wavelengths. Moreover, such a porous and reconfigurable stereo metasurface can be readily integrated with optofluidics, enabling broad applications in biological diagnostics, chiral pharmaceuticals, photobiology, etc.

**Fig. 11:**
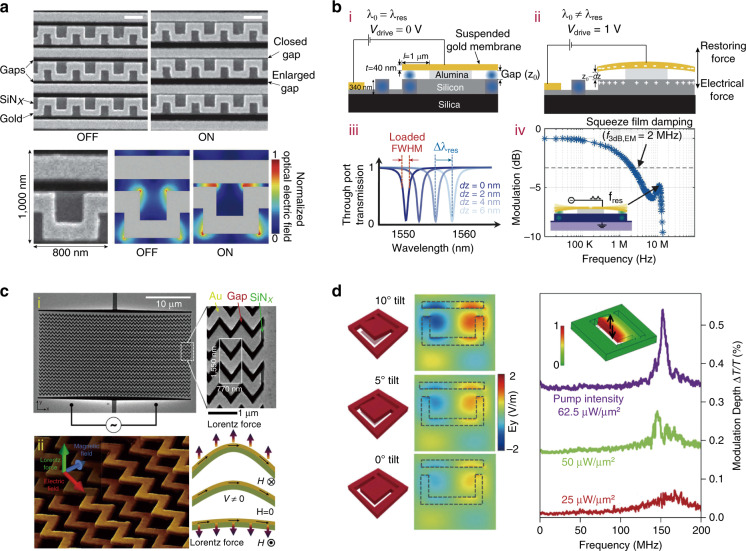
Potential schemes for high-speed reconfiguration. **a** Electromechanically reconfigurable metamaterial actuated by electrostatic forces between metallic “meander” and wire structures^112^. Scale bar: 500 nm. **b** (i, ii) Schematic of a hybrid photonic–plasmonic disc resonator, whose vertical gap can be tuned by electrostatic forces^113^. (iii, iv) The resonant spectra can be tuned with a speed of 12 MHz. **c** Magneto-electro-optical modulation scheme achieved in (i) a chevron metamaterial through (ii) Lorentz forces^114^. **d** Intensity-dependent optical force-actuated nano-optomechanical reconfiguration in a dielectric metamaterial^115^. Images reprinted with permission from: **a** ref. ^112^, **c** ref. ^114^ from Springer Nature; **b** ref. ^113^; **d** ref. ^115^ from AIP

To date, most of the kirigami/origami-based reconfiguration studies are focussed on the exploration of new tuning approaches and new physical phenomena, with little consideration of the slow modulation speed. Since high-speed reconfiguration is highly desirable in device applications, here several potential strategies with high-speed structural deformation are discussed, which are helpful for actuating kirigami/origami in a rapid manner. The first potential scheme is electrical reconfiguration. For example, as shown in Fig. 11a, Ou et al.^112^ developed an electromechanically reconfigurable metamaterial consisting of a metallic “meander” and a metallic wire, which were supported by silicon nitride strings and separated by an air gap. Upon application of a few volts to the neighbouring strings (conductive “meander” and wire structures), an attractive electrostatic force was induced, which moved the strings in the horizontal plane and closed the gap between them. This process could strongly affect the plasmonic mode of the meander pattern, allowing reversible modulation of the transmission and reflection at a high frequency of 1.6 MHz. More recently, Haffner et al.^113^ reported a hybrid photonic–plasmonic opto-electro-mechanical system (Fig. 11b) consisting of a thin gold membrane partially suspended above a silicon disc, which formed a vertical air gap that could be modulated by utilizing the electrostatic force under an applied voltage. In this way, the gold membrane bending (*dz*) could induce a resonance shift by changing the mode index, and the modulation speed could be as high as 12 MHz. More importantly, such a demonstration has challenged the common presumption that opto-electro-mechanics is a slow and bulky technology that requires high driving voltages.

The second potential scheme is to actuate kirigami/origami by utilizing magneto-electric-optical effects. In this aspect, Valente et al.^114^ demonstrated a large reciprocal magneto-optical effect in an artificial reconfigurable chevron nanowire structure fabricated on an elastic nanomembrane, which was driven by Lorentz and thermal forces, as shown in Fig. 11c. Fast reversible magneto-electro-optical modulation (resonant at 200 kHz) was observed when the metamaterial was placed under a fraction of a volt and in a fraction-of-a-tesla magnetic field. Last but not least, as another potential scheme, optical forces can induce even higher modulation speeds in nano-optomechanical systems. As presented in Fig. 11d, a nano-optomechanical dielectric metamaterial was demonstrated by Karvounis et al.^115^ by harnessing the large optomechanical nonlinearities in a nanostructured silicon membrane. By utilizing the resonance-enhanced optical forces within the metamaterials, the tilt angle of the cantilever within the metamolecules can be tuned, causing modulation of the probe signals. As the pump intensity was increased, the observed optomechanical resonance correspondingly grew and reached a high frequency of 152 MHz^115^.

## Conclusions and perspectives

In summary, we have comprehensively reviewed kirigami/origami as a new platform for advanced 3D microfabrication/nanofabrication. Various stimuli of kirigami/origami, including capillary forces, residual stress, mechanical stress, responsive forces, and FIB irradiation-induced stress, and their basic working principles in the microscale/nanoscale region are introduced and discussed. The progress in microscale/nanoscale kirigami/origami for biological and optical applications, as well as the potential to reshape 2D materials, construct metasurfaces, and realize optical reconfiguration, are briefly discussed. It can be seen that the advanced kirigami/origami provides an easily accessible approach for modulating the mechanical, electrical, magnetic, and optical properties of existing materials, with remarkable flexibility, diversity, functionality, generality, and reconfigurability. These key features clearly differentiate the facile kirigami/origami from other complicated 3D nanofabrication methods and make this new paradigm technique unique and promising for solving many difficult problems in practical application of microdevices/nanodevices. First, flexible 2D-to-3D transformation is accomplished in an automated manner, which greatly simplifies the fabrication difficulties of complex 3D nanostructures. Second, the diversity is enriched by the unprecedented 3D geometries enabled by kirigami/origami, which are beyond the existing geometries obtainable by conventional layer-by-layer and translational fabrication. Third, the functionality is greatly extended by the versatile 3D nanogeometries, which induce exotic physical characteristics and intrinsic behaviour, such as the giant optical chirality caused by 3D nanotwists. Fourth, the generality is reflected by the deformable configurations that are universally effective from macroscopic paper kirigami/origami to atomically thin graphene. Finally, the reconfigurability represents one of the most promising potential features, enabling competitiveness with nanophotonic reconfiguration, and can bring about important applications, such as novel state-of-the-art nano-opto-electro-mechanical (NOEMS) systems^116^.

Meanwhile, research on microscale/nanoscale kirigami/origami has only been initiated in the past decade and still faces a few limitations and challenges. First, the nanoscale “folding” mechanisms, i.e. the stimuli of the transformations, are limited to a few strategies in laboratories. Widely applicable and inexpensive transformation schemes, such as electrostatic force-induced in-plane and out-of-plane displacement, are needed to stimulate increasingly extensive interest and practical applications. Second, on-chip, large-scale, and integrable microscale/nanoscale kirigami/origami, which is desirable for device-level applications, has yet to be explored. For example, the standard UV lithography and CMOS techniques, as well as the emerging material platforms, might be helpful for the development of new methodologies. Third, the current kirigami/origami designs at the microscale/nanoscale mostly come from empirical and phenomenological experience, and the resulting geometries are still very primitive and limited by one’s normal expectations and imagination. Advanced and extraordinary kirigami/origami designs based on analytical methodologies and inverse principles could stimulate more new physics and applications but remain unexplored at the microscale/nanoscale. Finally, while a few strategies have been demonstrated for the reconfiguration in microscale/nanoscale kirigami/origami, fast and accurate optical reconfiguration is still a great challenge. Compared with surface tension forces that are normally slow in modulation, on-chip electrostatic and magnetic forces^117^ may provide competitive solutions to this issue but require profound investigations.

Therefore, it can be naturally expected that when these challenges are met and the advantages are fully adopted, microscale/nanoscale kirigami/origami will greatly innovate the regime of 3D microfabrication/nanofabrication. Unprecedented physical characteristics and extensive functional applications can be achieved in the wide areas of optics, physics, biology, chemistry, and engineering. These new-concept technologies, with breakthrough prototypes, could provide useful solutions for novel LIDAR/LADAR systems, high-speed DMD chips, high-resolution spatial optical modulators, integrated optical reconfiguration chips, ultra-sensitive biomedical sensors, on-chip biomedical diagnosis devices, and the emerging NOEMS systems that are promising for the modern industries of communication, sensing, and quantum information processing^116^.
